# Two-Year Hydrocarbon Variations in Sediments of the Ring of Cenotes, Yucatan, Mexico

**DOI:** 10.1007/s00128-025-04040-x

**Published:** 2025-04-21

**Authors:** Flor Arcega-Cabrera, José Andrés Martínez-Trejo, Elsa Noreña-Barroso, Gabriela Rodríguez-Fuentes, Ismael Oceguera-Vargas

**Affiliations:** 1 Unidad de Química en Sisal, Facultad de Química, Universidad Nacional Autónoma de México. Puerto de Abrigo S/N, 97355 Sisal, Yucatan Mexico; 2 Posgrado en Ciencias del Mar y Limnología, Universidad Nacional Autónoma de México, Avenida Ciudad Universitaria 3000, C.P. 04510 Mexico City, Coyoacán Mexico

**Keywords:** Polycyclic aromatic hydrocarbons, Sinkholes, Yucatan, Carbon preference index

## Abstract

Pollutant input to groundwater could result in karstic aquifers water quality degradation. In the karstic aquifer of Yucatan several anthropogenic activities take place without water sanitation or waste management, thus, these activities could be a source of contaminants to sinkholes (locally known as “cenotes”), which are the most relevant features in the karstic platform. In this context, this study monitored total n-alkanes (TAL) and polycyclic aromatic hydrocarbons (PAHs) in sediments of sinkholes at the ring of cenotes in Yucatan over a two year full hydrologic cycle (rainy-dry-rainy-dry seasons). Total PAHs measured in this study ranged from 1.7 to 1450 ng/g, and from 0.01 to 7520.8 ng/g for TAL. Spatially significant variations were only found between the eastern and central zones in dry season, probably as result of the environmental and hydrological changes. Main probable origin of hydrocarbons was found to be pyrogenic, resulting from local customs such as constant garbage burning and seasonal farmfields burning. Although PAHs did not show concentrations of concern yet, current socioeconomic development and no water sanitation envisages future threats to the karstic aquifer water quality for human supply.

## Introduction

Aquifers embedded in highly karstified carbonated rocks are often highly vulnerable to pollution, especially in developing countries where water sanitation and waste management are not a priority (Arcega-Cabrera et al. 2014, 2021b). Among the pollutants produced by anthropogenic activities, hydrocarbons are of high concern as result of their toxic properties (Hussein et al. 2016). Hydrocarbons are classified for study in aromatics and aliphatics on the basis of their sources and structural properties (Arcega-Cabrera-Dótor Almazán 2021a). Polycyclic aromatic hydrocarbons (PAHs), a significant subclass of aromatic compounds, are particularly notable because most of the PAHs are anthropogenic (Howsam-Jones 1998; Jessica 2021), although some come from natural sources (Capaccioni et al. 1995). PAHs are reported to be toxic, persistent, and can bioaccumulate and be transported in the air over long distances (Mehdi et al. 2022). The US EPA has established a list of 16 priority PAHs (based on toxicity, frequency of occurrence, potential for human exposure, and available information), of which seven are considered probable carcinogens to humans (ATSDR 1995; US EPA 2001).

Identifying the source of hydrocarbons (biogenic, diagenetic, petrogenic, or pyrogenic (Peters et al. 2005; Boehm 2006) could help in proposing pollution mitigation actions. The Carbon Preference Index (CPI), a concentration ratio between odd-carbon atom and even-carbon atom n-alkanes, can be used to assess whether hydrocarbons have a biogenic (predominance odd-carbon atom alkanes, CPI > 1) or petrogenic origin (when the CPI is close to 1) (Liu et al. 2009; Vane et al. 2014); PAH diagnostic ratios can also be used to evaluate pyrolytic and petrogenic origins of PAHs in sediments (Tobiszewski and Namiesnik 2012). PAHs are associated with organic matter in soils and sediments this promotes their sorption to particulates that could accumulate in sediments (McGrath et al. 2019; Kariyawasam et al. 2022). In this context, this study aimed to investigate two-year space and time PAH and total-n-alkanes (TAL)variations in sediments and the probable origin of hydrocarbons in the ring of cenotes area, a site of high economic and environmental relevance (Beddows et al. 2008), where groundwater is the only water source available for potable and anthropogenic uses.

## Methodology

**Fig. 1:**
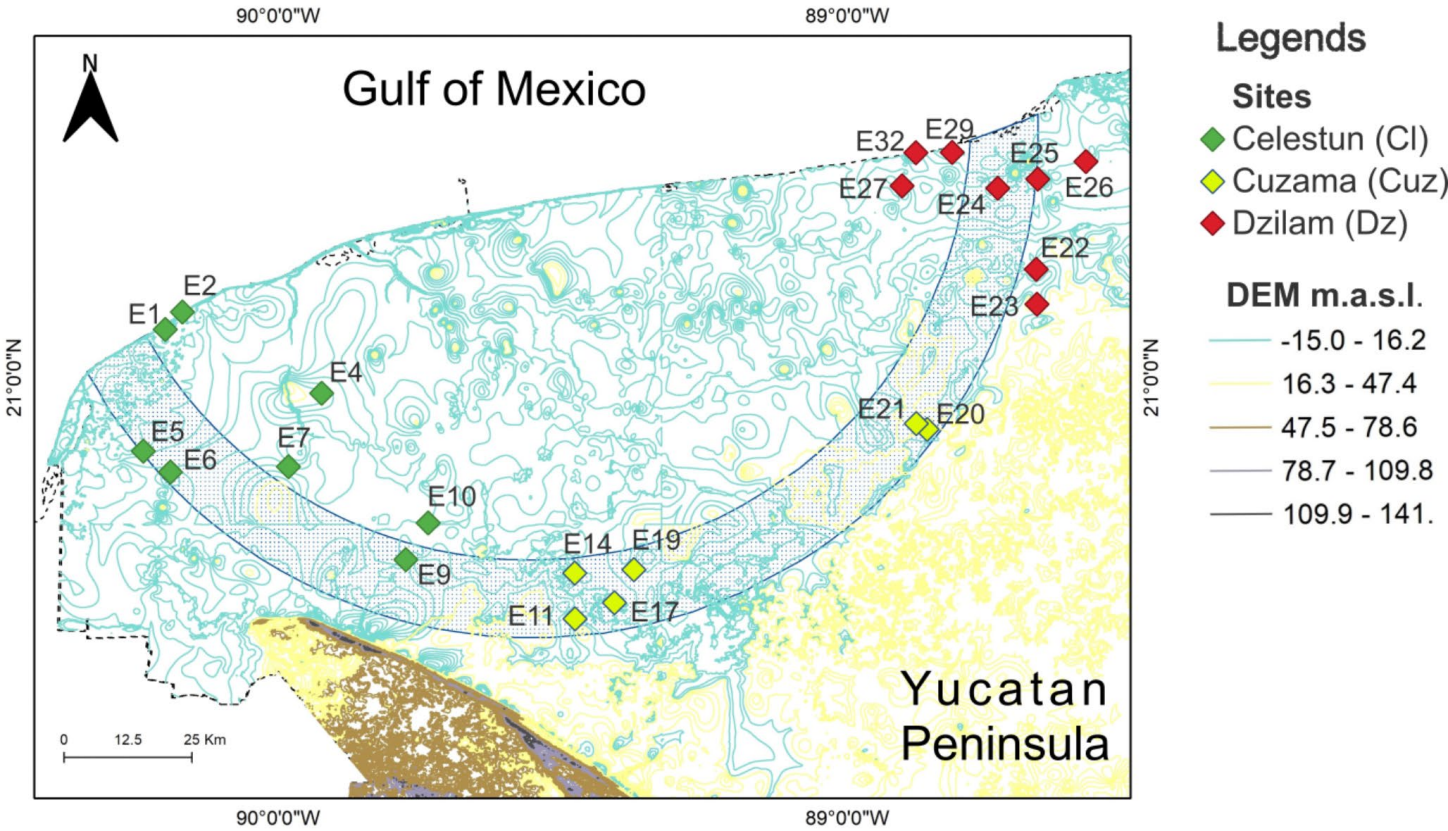
Site map of the Ring of Cenotes (Yucatan, Mexico) includes sampling stations for Celestun (green), Cuzama (yellow), and Dzilam (red) with elevation countour lines (DEM)

This study took place in Yucatan, Mexico, in the geohydrological reserve denominated “Ring of cenotes” (RC), a semicircular area with a high density of sinkholes. The climate in the area is tropical, with a mean temperature of 28º C and total annual precipitation is 1007 mm (CONAGUA, 2023). There is a marked rainy season (June-September), a “Nortes” season characterized by strong winds from the north (October-December), and a dry season (December-May) (Kantun et al. 2018, Metcalfe et al. 2020). Twenty-three sinkholes located in the RC were selected (Table 1 supplementary material) under the following criteria: safe and free access all year long and wide geographic distribution where human settlements and economic activities were present (Arcega-Cabrera et al. 2014). Sampled sites are shown in Fig. 1, the name, sinkhole type, anthropogenic activities, and zone are provided in Table 1 of the supplementary material. Sinkholes were sampled for a two year full hydrologic cycle (rainy-dry-rainy-dry seasons) from September 2012 to May 2014.

In these sites, surface sediments (ca. first 3 cm of the sedimentary column) were collected and samples were bagged and preserved at − 4ºC until analysis. Then, samples were freeze-dried and sieved through a Fieldmaster series of sieves. Then, the weight of the < 63 μm fraction was measured using an Ohaus scale with a precision of ± 0.01 g, and the percentage of this fraction in the total sample was calculated, ranging between 5 and 33%. The percentage of organic matter (%OM) was determined using the wet oxidation method (Walkley and Black 1934). Hydrocarbon analysis was performed based on the analytical procedure described in Kantun et al. (2018) with some modifications, performing an ultrasound-assisted extraction (USE) and fractionation (aliphatics and aromatics) by column chromatography on activated silica gel.

Gas chromatography coupled to mass spectrometry (GC-MS) and gas chromatography with flame ionization detector (GC-FID) techniques were used to quantify PAHs and n-alkanes, respectively. For full method conditions please refer to the supplementary material (Section A and Tables 3 and 4). PAH concentrations were separated into light polyaromatic hydrocarbons (LPAH) with two or three rings (acenaphtylene, acenaphtene, fluorene, phenantrene and anthracene) heavy polyaromatic hydrocarbons (HPAH) with more than four rings (fluoranthrene, pyrene benzo(a)anthracene, chrysene benzo(b)fluoranthene, benzo(a)pyrene, indeno(1,2,3-cd)pyrene, dibenzo(a, h)anthracene, benzo(g, h,i)perylene). Due to the number of rings in PAHs, light molecular weight PAHs tend to volatilize from the water, while heavier PAHs are more likely to settle out with the sediments and co-precipitate with particulate matter, leading to their accumulation in the sediment (Patel et al. 2020).

To identify sources for the PAHs detected in sediments we analyzed the following PAH concentration ratios: (a) petrogenic origin; anthracene/(anthracene + phenantrene) < 0.1, fluoranthene/(fluoranthene + pyrene) < 0.4 were mainly from petroleum contamination, and fluoranthene/(fluoranthene + pyrene) > 0.4 were mainly from combustion of petroleum (pyrogenic); and (b) pyrogenic origin; anthracene/(anthracene + phenantrene) > 0.1 were typical of combustion source, and fluoranthene/(fluoranthene + pyrene) > 0.5 were mainly from combustion of grass, wood and coal (Li et al. 2006; Liu et al. 2009). CPI was calculated to asses potential biogenic or petrogenic origin of aliphatic hydrocarbons.

Data were transformed using the function Log(x + 1) and normalized before analysis (Arcega-Cabrera et al. 2014). We used a model with three fixed factors: (i) cycle (2 levels; first or second year), (ii) zone (3 levels; Cl, Cuz or Dz), and (iii) season (2 levels; dry or rainy). Results were statistically evaluated with PERMANOVA with 9999 random permutations of the residuals under the reduced model, and principal coordinates analysis (PCoA) was done to visualize data (Anderson 2001). All statistical analysis and graphic representations of results were performed using Prime 7 with PERMANOVA add-on (Anderson et al. 2008).

## Results and Discussion

Several anthropogenic activities occur along the RC, which can produce and release different hydrocarbons (Arcega-Cabrera et al. 2014; Derrien et al. 2015). PAH concentrations measured in this study (Table 2 supplementary material) ranged from 1.7 to 1450 ng/g, with the mean concentrations representing data from the two years sampled. The concentrations were 86.8 ± 194.2 ng/g (mean ± standard deviation) for HPAH and ranged from 0.1 to 704.2 ng/g, with a mean of 144.5 ± 177.5 ng/g (mean ± standard deviation) for LPAH. According to the classification of Baumard et al. (1998), which analyzes PAH concentrations in aquatic environments and historical data in marine organisms relative to sediments and trophic levels, LPAH levels indicate low to medium pollution. Higher concentrations were observed during the dry season of the first year. A high level of HPAH was found at site E22 during the dry season of the second year. In pristine aquifers, PAH concentrations are low, with levels < 0.05 ng/g (Bhardwaj et al. 2021), in water bodies with urban or anthropogenic influence maximum PAH concentrations are > 1000 ng/g (Liu et al. 2009; Du and Jing 2018; Li et al. 2022; Yao et al. 2023); compared to other karstic aquifers in the peninsula (Quintana Roo, Mexico), which reported very high concentrations (> 5000 ng/g) indicating a direct influence of anthropogenic contamination primarily from tourist activity (Metcalfe et al. 2011; Medina-Moreno et al. 2014; Lizardi-Jiménez et al. 2015; Moreno-Pérez et al. 2021), the sites studied in this research have lower concentrations. This suggests that, although the sites are not pristine, they are less impacted by direct anthropogenic activities.

Regarding TAL, most values ranged (0.01–7520.8 ng/g) except for a very high concentration measured in site E26 (Rancho Santa Elena) in the second year dry season. Odd-carbon atom alkanes from n-C21 to n-C35 are linked to a biological source (plants and organic matter degradation) (Calva et al. 2005; Pu et al. 2017); toxic effects were not observed for aliphatic hydrocarbons in aquifers at concentrations below approximately 5000 ng/g (Parkerton et al. 2021). Since the aliphatic hydrocarbon concentration at site E27 is higher, ecotoxicological assays should be conducted to confirm potential toxic effects at this site.

The %OM values are below and above (4.3–9.5%) than the range of the reported by Derrien et al. (2015) ( (6.3–15.9%) and %FINAS are in the range of the reported by Brown et al. (2014) (3–11%) for Yucatan’s cenotes; the mean particle sizes in sinkholes ranged from medium sands to lime mud category, therefore sinkhole sediments could be accumulating contaminants in this fine grained-high organic content sediments (Arcega-Cabrera at al. 2014). Results of all the measured and calculated variables are shown at Table 1 in the supplementary material.

**Fig. 2:**
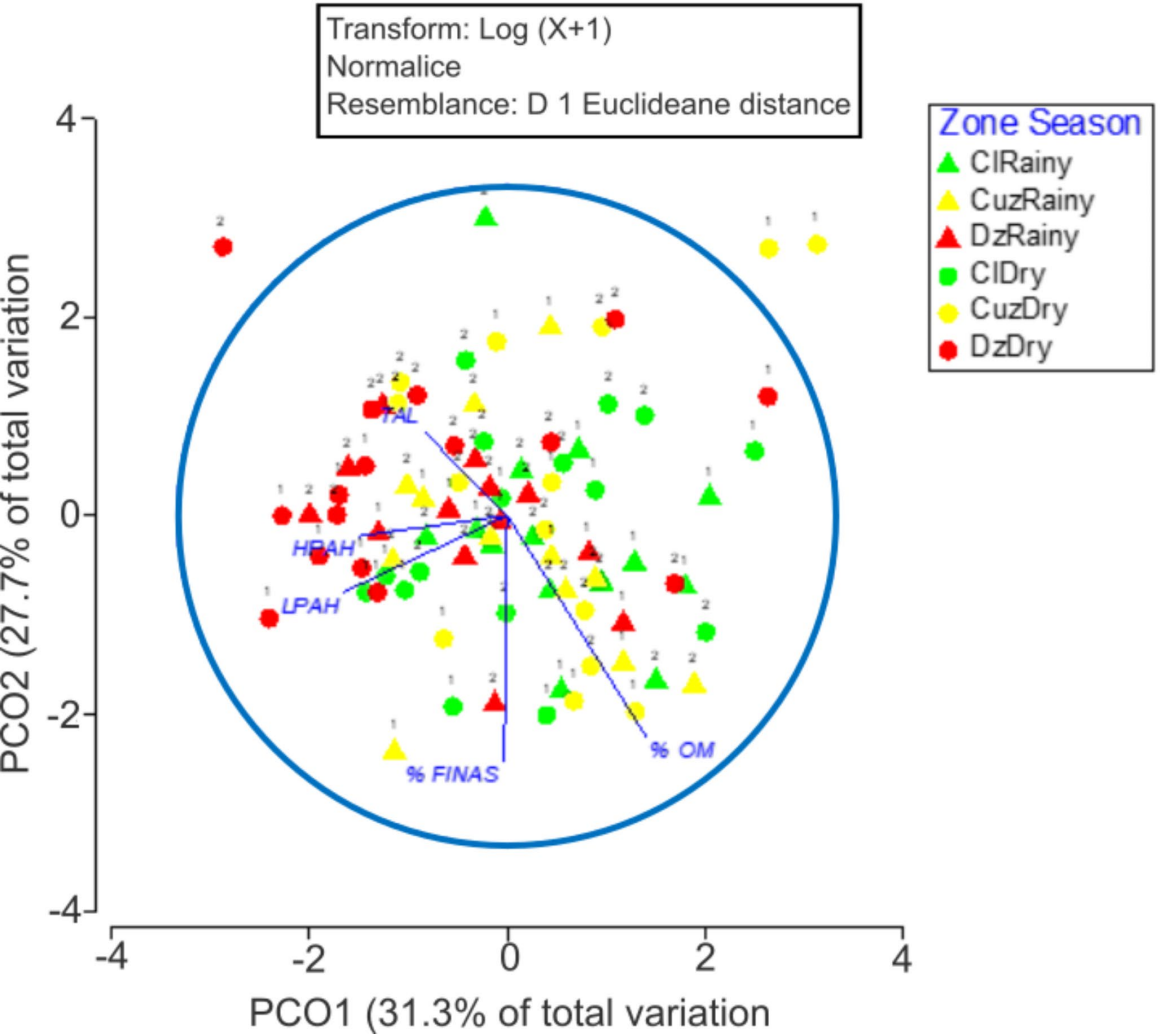
Principal coordinate analysis (PCO) of two-year (year 1 or 2) sampling of hydrocarbons, organic matter and percentage of fine sediments (%FINAS) in cenotes of Yucatan, Mexico in three different zones, Celestun (Cl, green), Cuzamá (Cuz, yellow) and Dzilam (Dz, red) in the dry (circles) and rainy season (triangles)

The PCoA was applied to the TAL concentrations, low molecular weight PAHs (LPAH), high molecular weight PAHs (HPAH), percentage of fine sediments (%FINAS), and the percentage of organic matter (%OM), after two cycles of samplings it explained (58.92%) of the total variation by two principal coordinates (Fig. 2).

**Fig. 3:**
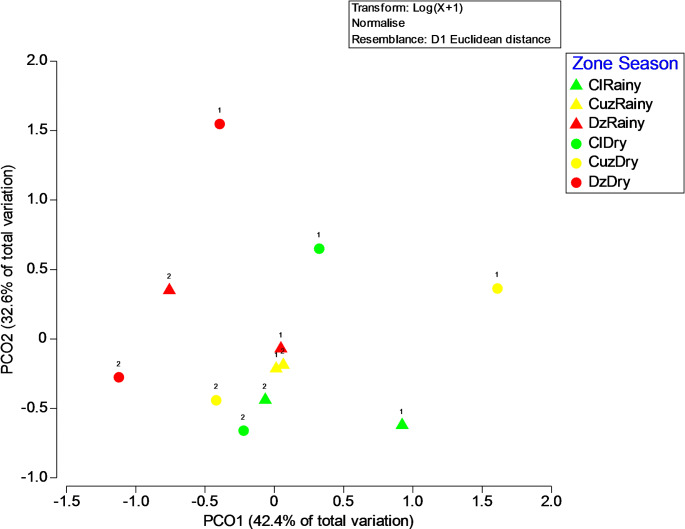
Principal coordinate analysis (PCO) of the centrioids of two-year (year 1 or 2) sampling of hydrocarbons, organic matter and percentage of fine sediments (%FINAS) in cenotes of Yucatan, Mexico in three different zones, Celestun (Cl, green), Cuzamá (Cuz, yellow) and Dzilam (Dz, red) in the dry (circles) and rainy season (triangles)

The eigenvectors that contributed most to sample separation in the first coordinate was LPAH (− 0.50). The second principal coordinate %FINAS (− 0.75) was the eigenvectors that contributed most to the separation of samples. The PERMANOVA showed a significant interaction between factors season and year (pseudo-F = 3.1; *p* = 0.013, 9953 unique permutations), indicating that the response varied between years and seasons. Post hoc paired comparisons indicated that the measured concentrations of these variables have significant differences between Cuz and Dz in the dry season of the first year. This behavior is easier to visualize in Fig. 3, where the centroids of these factors are graphed. Cuz values in the first year dry season are characterized by lower values of PAHs and TAL and higher percentages of organic matter and fine sediments. The rest of the paired comparisons per cycle, season, and sites were non-statistically significant.

A spatial difference was found in the first year dry season between Cuz and Dz. These variations could be related to the hydrogeological context and specific anthropogenic sources (Arcega-Cabrera et al. 2021b). PAH inputs to the sinkholes increases during the dry season, when groundwater flow diminishes, and some PAHs, adsorbed to the particulate matter, settle down and slightly increase the sediment’s concentration. Contrary, during the rainy season groundwater flux increases and sedimentation rate diminishes. The relatively low concentrations measured in the sediments of the cenotes could result from the fact that once PAHs are released into the environment, they are prone to a wide variety of processes, including evaporation, dissolution, dispersion, emulsification, adsorption on suspended materials, microbial degradation, photo-oxidation, and interaction among the contaminants and sediments (Stogiannidis and Laane 2015). PAHs were found to be pyrogenic and petrogenic; the CPI was biogenic and petrogenic (Li et al. 2006; Liu et al. 2009). A pyrogenic origin was expected as the result of the customary practices, such as burning agricultural fields before sowing new crops and daily garbage burning. Also, the prevalence of wildfires increases during the dry season. Besides, in second year dry season there was a negative temperature anomaly (1.2 °C lower than the previous one - Meteoblue 2022).

Accordingly, there was a positive precipitation anomaly (33 mm in the second year dry season, contrary to the − 0.6 mm in the previous one); then, as reported by Arcega-Cabrera et al. (2014, 2021b) the amount of precipitation in a karstic open aquifer will have a significant impact on the input from point and non-point sources, and also on the fate of pollutants (dispersion/accumulation in a given flow direction) in groundwater; and the amount of precipitation changes between hydrological seasons, watershed regions, and years (PHR 2021–2024). The results in the second year could be related with these previously mentioned changes since the environmental conditions in the dry season are like the ones in rainy season, with a lower temperature and a higher precipitation than the mean one expected for dry season, therefore, this explains the similar tendency in the data behavior observed for the rainy season and the anomalous dry season. Besides, given the former, an increase in the hydrocarbon’s wet precipitation to the sinkholes in the anomalous dry season, especially in the open cenotes, like the ones in Dz that presented higher PAH concentrations than Cuz, could be anticipated. According to Stogiannidis and Laane (2015), anthropogenic PAHs resulting from the utilization of petroleum products and incomplete combustion of fossil fuels, biofuels, or other forms of organic matter far exceed natural sources. Therefore, it is probable that PAHs also have an anthropogenic source. Regarding the Carbon Preference Index (CPI) (Arcega-Cabrera and Dótor-Almazán 2021), the sites show a biogenic CPI, indicating an autochthonous (local) origin. In contrast, a petrogenic CPI would indicate an allochthonous (external) origin. Biogenic hydrocarbons are mainly formed by biological processes from plants or animals such as algae, phytoplankton, and microorganisms. In general, LPAHs show a predominance of petrogenic origin, (petroleum products, incomplete combustion of fossil fuels, and natural diagenesis). Conversely, HPAHs exhibit a predominance of pyrogenic origin, primarily formed by the incomplete combustion of organic materials. (Du and Jing 2018). In our study, LPAHs had higher concentrations than HPAHs, except for one site (E22 during dry season), this suggests that the petrogenic origin dominted in the Ring of Cenotes; however, using other ratios as Fla/(Fla + Pyr) aids to distinguish more precisely between petrogenic and pyrogenic (Du and Jing 2018; Queb-Suarez et al. 2022), where it implies a mixed origen. Total PAH values in open cenotes were 92.5 ± 159.4 ng/g during the rainy season and 167.6 ± 242.9 ng/g in the dry season, while in closed cenotes, they were 65.1 ± 95.9 ng/g during rainy season and 119.7 ± 136.9 ng/g during dry season. These results suggest open cenotes are more vulnerable to pollutants. Contrary to expectations, values were higher during the dry season, likely due to increased anthropogenic activities in summer (SEFOTUR, 2024). For site E22 during dry season, the PAH origin is pyrogenic (predominance of, HPAH), and social conditions suggest burning of garbage o agricultural fields. Site E26 exposed a very high TAL concentration, concentrations above 5000 ng/g implies contamination and toxic risk (Pu et al. 2017; Parkerton et al. 2021), site E26 is a sinkhole in a ranch that is used for agricultural and tourism purposes, also a pyrogenic origin could be explained by the extensive practice of burning garbage and fields. The months with anomalously dry behavior (May 2013 with only 72 mm when mean precipitation is 95 mm) showed a petrogenic origin of hydrocarbons contrary to the pyrogenic and mix origin founded in the years with mean to high precipitation (September 2012:141.4 mm; October 2013:177.1 mm and May 2014:175.1 mm). This could be result of (1) increased rain transport and deposit of the hydrocarbons present in the atmosphere (pyrogenic-Du and Jing 2018; Queb-Suarez et al. 2022), and (2) in low precipitation season the increase of groundwater transported and deposited hydrocarbons in the sinkhole sediment (petrogenic from e.g. oil dumping by industrial activities, car workshops, etc- Howsam and Jones., 1998).

## Conclusions

Although PAH concentrations found in the ring of cenotes ranged from low to medium levels, there is a high probability that unorganized urban development and the incorrect residues and wastewater treatment and disposal could promote increasing concentrations in the future, as has been reported in Quintana Roo’s aquifers (Metcalfe et al. 2011; Medina-Moreno et al. 2014; Moreno-Pérez et al. 2021). Spatial variations and the presence of pyrogenic hydrocarbons may result of the customary practices, such as burning agricultural fields before sowing new crops and urban residues daily burning. Temporal variations are the result of the environmental and hydrological changes, but also the type of cenote plays an important role in PAH inputs, open-type cenotes exposed to external inputs due to their direct connection with surface runoff, making them more vulnerable to the entry of hydrocarbons and other pollutants. This is particularly evident during periods of intensified anthropogenic activity, which may contribute to higher contaminant concentrations in sediments. High concentrations of HPAH (site E22) and TAL (site E26) have similar orgins, both are pyrogenic, this suggested that the high concentration registered comes from a direct input of an excessive and continuos burning garbage or agricultural fields, these concentrations can increase if human activity increases such as tourism. Also, the volume of precipitation will be determinant in the probable origin of the hydrocarbons present in the sediments (pyrogenic/mixed vs. petrogenic) as result of changes in the main entrance way, being either atmospheric or groundwater.

Cenotes are sensitive systems prone to pollution given their karstic context and, in many developing countries, low environmental impact socioeconomic development is not a priority and promotes karstic aquifers contamination. The results of this study showed the relevance of constant monitoring in karstic sites that consider not only the probable in situ contaminant sources, but also the main hydrological variations as well as the customary practices, that promote spatial and time variations that could be of concern when groundwater is for human use.
